# The Influence of Vicious Systemic States on the Teeth

**Published:** 1876-03

**Authors:** D. C. Hawxhurst


### THE INFLUENCE OF VICIOUS SYSTEMIC STATES ON THE TEETH.

BY D. C. HAWXHURST.

*Second part of a paper read before the Michigan State Dental Association, October, 1875.*

I can not pass this topic with out a few words about the effects of constitutional vices and disorders on the teeth.

Hutchinson it was who first pointed out that inherited syphilis could produce a variety of mal-formations of the teeth. This is more marked in the incisors, which are short, narrowedged and marked by curved notches or crescent-like grooves. Though often trivial, these points of defect do sometimes render the teeth unsightly, and make them an easy prey to decay. That the dentist should be able to recognize this state of things in his little patient and direct appropriate treatment during the gestation of subsequent children, needs only to be mentioned to be believed.

I pass, unconsidered, those frightful lesions which syphilis often produces in the face, to consider its less obvious effects on nutrition.

It will often embarrass the efforts of the practitioner by impairing nutrition in the tissues on which he operates, and by weakening vital force. Thus he will not find the same readiness of growth and repair in the tissues; he can not rely so much upon the *vis medicatrix natura*, and will not, in cases wherein the constitution has this taint, undertake as bold operations as he would where the vital force is abundant and normal. The large number of patients who come to us with this diathesis, present no certain marks by which a man not medically educated, could detect the *latent vice of eonstitution.*

The right action and strength of the vital forces of the tissue-elements seem to be impaired in persons thus afflicted, and the man who promises to his patient the usual results of operations will mislead him.

It being my object here to show that the dental practitioner ought to be well-grounded in those fundamental sciences on which medicine is based, I do not dwell on this affection longer than to show that it is directly related to the practice of dentistry.

Peculiar conditions of the uterus have been known to cause severe tooth-ache; this is too common to require illustration. And the gravid uterus and its contents do often cause an abstraction of lime-salts from the teeth and periostea, resulting in rapid and devastating decay. Here we have a constitutional influence affecting the teeth, which will be best met by that practitioner who knows most about the physiology and pathology of gestation.

Quite as serious as the above in its effects on the teeth is the disorder known as mollities ossium or osteomalacia. The dental organs partake of the general constitutional vice and exhibit grave defects of structure. The disease being one of morbid nutrition due to a perversion of cell-action, its treatment has already baffled some of our profoundest pathologists. It predisposes the teeth to rapid decay. The best medical knowledge which this country or any other can afford us, will not teach us too much about this disease.

When we go back to the formation of the teeth, we find their tissues developing under various states of health, and under the influence of injurious constitutional vices; we find their structure modified by impaired nutrition, their chemical constitution degraded by the presence of disturbing impurities in the humors. And all this, if not changed under the advice of the judicious dental surgeon, is to pave the way for early loss of the organs. The dentist who would do his whole duty to these cases must solve many a knotty problem of depraved nutrition and deal with many a mysterious aberation from normal vital action.

The time is coming, when prospective mothers will rectify their morbid physical states under correct advice and appropriate treatment; they will seek advice in behalf of the teeth of their children yet unborn.

And after the period of gestation is over, and lactation is commenced, the unerupted teeth will be constantly remembered and an effort made to secure their right organization.

Often the practitioner will treat the little patient's teeth through the mother's breast. Especially where the mother's milk and blood are deficient in the salts that harden the teeth, there will be an indication for modifying the diet or instituting appropriate medication.

Direct treatment of the child itself, begins when it is able to digest food other than the milk, and should extend beyond second dentition, eradicating every constitutional vice, and if that can not be done, shielding the teeth from its influence.

To do this wisely and well will be simply impossible to those who practice their profession as a handicraft. Indeed the wisest physician will need the whole resources of his art if he would do his whole duty to these patients.

There are many states of the system that must cause embarrassment to the dental surgeon and the proper handling of which must call out all the resources of his medical learning—the inflammatory tendency, the hæmorrhagic diathesis, the anæmic state which will harass him with neuralgia and dying pulps, the hysterical state which will make anæsthesia a terror to him.

It would be a great task to treat of all the morbid physical states that are capable of causing, predisposing to, or exacerbating disease in the teeth; it would be to write a treatise. That they should be understood by the man who has important cases on his hands that he wants to be successful in curing, is not a matter of question.

Every blood poison and whatever can effect the composition of the blood must influence the dental tissue for good or ill. Every cause that can morbidly affect the cells that assimilate pabulum or interfere with normal innervation must act as a cause of defective dental tissue, if in action during the period of their formative development.

But I can not give more time to this topic. I believe that the average patient as presented at our offices is, in the words Garrettson, "only half treated if only locally treated." And I see no way to meet the imperative requirements of cases as they come to us except through a profound acquaintance with the medical sciences.

